# Controversies in Tropical Medicine and Hygiene

**DOI:** 10.4269/ajtmh.2012.12-0427

**Published:** 2012-08-01

**Authors:** Joseph M. Vinetz

**Affiliations:** Editor-in-Chief, American Journal of Tropical Medicine and Hygiene

*The American Journal of Tropical Medicine and Hygiene* publishes original research papers, reviews and commentaries on diverse diseases and research areas of global health importance. Diseases that take the highest human toll—malaria, tuberculosis, and other neglected tropical diseases—are often those where evidence is most limited regarding pathogenesis, diagnosis, treatment or vaccine-mediated protection. Where data are limited, controversy exists and subjective opinions dominate.

To foster open, vigorous debate that will guide our diverse fields toward greater insights and ultimately impact on human lives, the *Journal* is embarking on publishing debates of topics that have widespread clinical and public health importance. These debates will take place within a Point-Counterpoint format published in the front matter of the journal. We invite interested authors to discuss with the Editor ideas and subject areas that would be amenable to formal, written interactive debate. For areas of mutual interest, authors will be invited to write a focused essay (less than 1500 words) putting forth a particular, possibly provocative viewpoint on a topical subject.

Submissions will undergo peer-review and authors with contrasting points of view will be invited to respond. Manuscript drafts will be shared among authors and between sides to allow for full vigorous debate and to allow for responses to points that are raised. We look forward to engaging with our authors and readers on important controversies in global health, the resolution of which has public health and clinical implications.

